# A Single Infusion of Polyethylene Glycol-Coated Superparamagnetic Magnetite Nanoparticles Alters Differently the Expressions of Genes Involved in Iron Metabolism in the Liver and Heart of Rats

**DOI:** 10.3390/pharmaceutics15051475

**Published:** 2023-05-12

**Authors:** Michal Kluknavsky, Andrea Micurova, Martin Skratek, Peter Balis, Monika Okuliarova, Jan Manka, Iveta Bernatova

**Affiliations:** 1 Centre of Experimental Medicine, Slovak Academy of Sciences, Institute of Normal and Pathological Physiology, 813 71 Bratislava, Slovakia; 2 Institute of Measurement Science, Slovak Academy of Sciences, 841 04 Bratislava, Slovakia; 3 Department of Animal Physiology and Ethology, Faculty of Natural Sciences, Comenius University, 842 15 Bratislava, Slovakia

**Keywords:** iron oxide nanoparticles, blood pressure, iron metabolism, oxidative stress, nitric oxide, magnetometry, NRF2

## Abstract

This study investigated genotype- and tissue-related differences in the biodistribution of superparamagnetic magnetite (Fe_3_O_4_) nanoparticles (IONs) into the heart and liver of normotensive Wistar Kyoto (WKY) and spontaneously hypertensive (SHR) rats after a single i.v. infusion of polyethylene glycol-coated IONs (~30 nm, 1mg Fe/kg) 100 min post-infusion. The effects of IONs on the expression of selected genes involved in the regulation of iron metabolism, including *Nos*, *Sod* and *Gpx4*, and their possible regulation by nuclear factor (erythroid-derived 2)-like 2 (NRF2, encoded by *Nfe2l2*) and iron-regulatory protein (encoded by *Irp1*) were investigated. In addition, superoxide and nitric oxide (NO) production were determined. Results showed reduced ION incorporations into tissues of SHR compared to WKY and in the hearts compared to the livers. IONs reduced plasma corticosterone levels and NO production in the livers of SHR. Elevated superoxide production was found only in ION-treated WKY. Results also showed differences in the regulation of iron metabolism on the gene level in the heart and liver. In the hearts, gene expressions of *Nos2*, *Nos3*, *Sod1*, *Sod2*, *Fpn*, *Tf*, *Dmt1* and *Fth1* correlated with *Irp1* but not with *Nfe2l2,* suggesting that their expression is regulated by mainly iron content. In the livers, expressions of *Nos2*, *Nos3*, *Sod2*, *Gpx4*, and *Dmt1* correlated with *Nfe2l2* but not with *Irp1*, suggesting a predominant effect of oxidative stress and/or NO.

## 1. Introduction

In organisms, iron absorption, storage and usage are highly regulated. Briefly, the main regulator of systemic iron homeostasis is hepcidin (HEPC, encoded by the *Hamp* gene)—a hormone primarily secreted by hepatocytes and, to a lesser extent, also in the heart [1] and other tissues, in response to iron loading. Hepcidin binds to ferroportin (FPN, encoded by the *Fpn1* gene), the iron exporter on the cell surface. This initiates FPN internalization and degradation resulting in a reduced efflux of iron from the enterocytes, macrophages and other peripheral cells into the circulation [2,3]. In circulation, iron is transported via the circulating transporter transferrin (TF, encoded by the *Tf* gene). After TF binding to transferrin receptor-1 (TFR1, encoded by the *Tfr1* gene) on the peripheral cells, transferrin-bound iron enters the cells in which iron accomplishes its biological functions. Non-transferrin-bound iron enters the cells via the divalent metal transporter (DMT1, encoded by the *Dmt1* gene) [4]. Intracellular iron metabolism is regulated predominantly through the iron-regulatory protein (IRP1 encoded by the *Irp1* gene) that binds to iron-responsive elements in specific mRNAs [5]. While iron absorption is highly regulated, the organism has no active mechanism to excrete the redundant iron; however, iron can be deposited in ferritin (FTH, encoded by the *Fth1* and *Ftl* genes) cores [6]. The absence of active excretion mechanisms can thus result in the cellular accumulation of iron and/or free iron can occur in circulation, while both can be cytotoxic.

Disorders in iron metabolism were found in many diseased states. Epidemiological studies provide evidence that elevated iron stores are a risk factor for developing cardiovascular and metabolic abnormalities [7]. On the other hand, low iron concentration was found in heart failure [8]. In addition, Seravalle et al. have shown that hepatic iron overload correlated positively with muscle sympathetic nerve activity in hypertensive patients, suggesting that hepatic iron overload may participate in increased cardiovascular risk [9]. There is also crosstalk between the liver and heart: liver dysfunction may induce cardiac disorders and vice versa [10], thus iron-overload-induced hepatic disorders may have a negative impact on the cardiovascular system.

Nowadays, iron oxide nanoparticles (IONs) are widely used in various areas, such as the cosmetic industry, biomedical research, medicine and others. In medicine and biomedical research, IONs can be used as contrast agents to improve magnetic resonance imaging, for cell labelling, targeted drug delivery, vascular tissue repair or for cancer treatment with hyperthermia [11]. Pharmacokinetics and biodistribution of IONs and other metal nanoparticles [12,13] and blood half-lives of IONs coated with different types of molecules in mammal models were reviewed previously [14]. The pharmacokinetics and half-life of IONs depend on various factors, such as size, shape and surface chemistries, and IONs can enter cells via phagocytosis and pinocytosis [14,15]. Under certain conditions (low pH in lysosomes, instability of ION coating or in the case of interactions with other substances), the ION coating may become unstable and the iron can be released. Free iron can cause oxidative stress due to the production of reactive oxygen species (ROS) in the superoxide-driven Fenton reaction, which consequently leads to peroxide radical formation, lipid oxidation and, in case of reduced glutathione peroxidase 4 (GPX4, encoded by the *Gpx4* gene) activity, iron-dependent cell death called ferroptosis [16,17]. On the other hand, following the elevation of ROS levels, cytoprotective pathways are activated, which are mediated via transcription nuclear factor (erythroid-derived 2)-like 2 (NRF2, encoded by the *Nfe2l2* gene) [18]. The *Nfe2l2* gene is constitutively expressed in all cell types, thus ensuring their prompt response to oxidative, inflammatory and metabolic stresses. Under normal conditions, the NRF2 protein has a rapid turnover with a half-life of about 20–30 min [19]. NRF2 translocation into the nucleus is elevated in the presence of elevated ROS as well as nitric oxide (NO) [20,21] and it induces the transcription of genes involved in antioxidant defence [22].

Another role of NRF2 is the iron-independent regulation of genes involved in iron metabolism, such as *Fth1*, *Ftl*, *Fpn1*, heme oxygenase 1 and others [18]. In addition, recent studies found that NRF2 indirectly controls the release of HEPC [23,24]. In conditions of oxidative stress, peroxisome proliferator-activated receptor gamma (PPARγ, encoded by *Pparg* gene) also plays an important regulatory role, which directly modulates the expression of superoxide dismutases (SOD1 and SOD2, encoded by the *Sod1* and *Sod2* genes), catalase [25] and inducible and endothelial NO synthases (NOS2 and NOS3, encoded by the *Nos2* and *Nos3* genes, respectively) [26,27]. A recent study also showed that i.v. administration of amino-modified IONs led to significantly increased *Hamp* mRNA in mice with non-alcoholic fatty liver disease [28]. In another study, TF saturation and TFR1 serum levels were increased along with increased *Hamp* expression and HEPC serum levels [29]. These findings support the hypothesis of changes in hepatic iron metabolism following the administration of IONs. In addition, the administration of IONs was also shown to interfere with the gene expression of NRF2 and nuclear receptor PPARγ [30,31].

In recent studies, we have shown that elevated BP was associated with alterations in the distribution of IONs compared to rats with normal blood pressure [13,32]. However, little is known about differences in iron metabolism and the incorporation of IONs into the heart and liver in hypertensive rats and whether and how IONs affect the expression of genes involved in iron metabolism, antioxidant defence and NO production in these organs after a single i.v. infusion, a condition that is relevant if IONs are used for diagnostic or therapeutic purposes in patients with various BP levels.

Thus, this study investigated the influence of a single intravenous infusion of IONs in the liver and the heart in normotensive Wistar Kyoto (WKY) and spontaneously hypertensive rats (SHR). We focused on genotype- and tissue-related differences in the biodistribution of IONs, superoxide and NO productions, gene expressions of the selected genes involved in iron metabolism as well as in genes involved in NO production and maintenance of redox balance in the liver and heart. We hypothesized that chronic elevation of blood pressure (hypertension) significantly alters the biodistribution of IONs and thus alters the expression of the above-mentioned genes, with more pronounced alterations in the liver than in the heart.

## 2. Materials and Methods

### 2.1. Animals and Treatment

In our study, 12–16 weeks old normotensive WKY and SHR male rats were used. They were fed with pelleted diet 1320, variant P (Altromin 1314, variant P; Altromin Spezialfutter, Lage, Germany), with reduced phytoestrogen content and defined iron content (192.51 mg/kg of diet). Rats had free access to food and water. The rats were held in standard conditions (humidity 45–65%; temperature 20–22 °C) with a regular rotation of light and darkness (12/12 h). Before the experiment, all of the rats had implanted catheters in the carotid artery for blood pressure (BP) and heart rate (HR) measurements and a catheter in the jugular vein for infusion of nanoparticles dispersed in physiological saline solution or saline alone (in the control rats). The rats were cannulated under anaesthesia (2.5–3% isoflurane) by the procedure described previously [33] one day before the experiment. The rats were divided into 2 groups in each genotype: control groups to which saline alone was administered (Cont, n = 6–7) and ION treated group (ION, n = 6–7) to which a dispersion of IONs in saline was administered.

Commercially available superparamagnetic PEG-coated IONs were purchased from Merck (previously Sigma-Aldrich) Bratislava, Slovakia (cat. No. 747408, PubChem SID 329765832, accessed on 18 March 2021). Coated IONs were chosen because bare IONs were shown to be toxic due to their ability to induce haemolysis, DNA breakdown and oxidative stress [34,35]. PEG was chosen as a suitable coating as it is a hydrophilic, biocompatible and neutral polymer that enhances the dispersion of IONs in aqueous media. Furthermore, PEGylation of nanoparticles has been demonstrated to reduce immunogenicity, liver uptake, hemotoxic effects and haemolysis, as well as increase the blood circulation time of nanoparticles in vivo [36,37,38]. IONs concentration was 1 mg Fe/mL and they were dispersed in water. The manufacturer declared the size of IONs confirmed by the transmission electron microscope was 28–32 nm, the zeta potential was −12 mV, the polydispersity index was 0.1 and the hydrodynamic size was approximately 45 nm. The exact properties of IONs used in this study were described previously [39]. IONs were autoclaved at 121 °C for 30 min and dispersed in sterile saline to reach a final dose of 1 mg of Fe/kg of body weight and administered in 10 min-lasting infusions into the jugular vein of rats. At the end of the experiment, i.e., 100 min after the IONs (or saline to control rats) administration, a small amount of blood was collected from the carotid artery catheter into ethylenediaminetetraacetic acid-coated test tubes to prepare plasma for determination of plasma corticosterone. Rats were then decapitated unconsciously after short exposure to CO_2_, and trunk blood was collected into heparinized test tubes for determination of plasma iron concentration. Then the hearts and livers were dissected for further analyses.

### 2.2. Blood Pressure and Heart Rate Determination

Before the experiment, the conscious rats were placed into a plastic box 27 × 14 × 9 cm in size, which allowed free movement. BP and HR were recorded with a sampling rate of 1 kHz via a catheter inserted into the carotid artery using the BP-recording device PowerLab system (ADInstruments, Bella Vista, Australia). BP and HR were recorded during the entire experiment, basal mean arterial pressure (MAP) and HR were calculated as the average values of a 200 sec segment between 16 and 20 min of the recording (about 10 min before IONs application) and end MAP and HR were calculated as the average values of a 200 sec segment between 138 and 142 min of the experiment (100 min after ION application) [32].

### 2.3. Determination of Plasma Corticosterone and Iron Concentrations

After collection, blood was centrifuged at 4 °C, 850× *g* for 10 min and plasma was kept at −80 °C until further analysis. Plasma corticosterone was determined in plasma samples using the Corticosterone Rat/Mouse ^125^I radioimmunoassay kit (DRG Instruments GmBH, Germany) according to the manufacturer’s instructions. All samples were measured in a single assay with the intra-assay variation coefficient of 4%. The assay sensitivity was 6.3 ng/mL [32].

Iron concentrations were determined in blood plasma from heparinized trunk blood in the accredited laboratory of Laboklin s.r.o. (Bratislava, Slovakia) using the standard laboratory methods [40].

### 2.4. Determinations of NOS Activity and Superoxide Production

Total nitric oxide synthase (NOS) activity was determined in the 20% of tissue homogenates (*w*:*v*) of the liver and left heart ventricle (LHV) as described previously [32]. Briefly, tissues were collected into ice-cold buffer (0.05 mol/L Tris–HCl, pH 7.4, containing 1% protease inhibitor cocktail, homogenized 4 °C and centrifuged (4 °C, 10 min, 3000× *g*). After centrifugation, NO synthase activity was determined in the supernatants by the conversion of [^3^H]-L-arginine to [^3^H]-L-citrulline. NO synthase activity was expressed as pkat per gram of protein. The protein concentration was determined using the Lowry method [41].

Superoxide production was determined in approximately 15 mg tissue of the liver and LHV by determination of lucigenin-enhanced chemiluminescence as described previously [42,43]. The results were expressed as cpm/mg of tissue.

### 2.5. Determination of the Relative Content of Biogenic Iron and ION-Originated Iron Content in Plasma, Liver and Heart

To determine the relative content of biogenic iron and iron-containing compounds in the liver and LHV samples, we used a Quantum Design MPMS-XL 7AC (SQUID) magnetometer with a reciprocating sample operation option with a sensitivity of 10^−11^ Am^2^. The advantage of this method is its ability to determine all the iron forms in a small sample of biological material with high sensitivity [44,45,46]. It also allows us to distinguish biogenic iron from that originated from IONs based on their different magnetic properties [39].

Samples of the liver and LHV were dissected using ceramic scissors and forceps, frozen immediately in liquid nitrogen and kept at −80 °C until further analysis. Before the analysis, the tissues were defrosted and cut using the cylindrical shaped instrument to obtain repeatedly the same shape of all samples with d = ~4.5 mm as described previously [39]. These were vacuum dried and inserted into a plastic measuring straw. For the determination of the relative content of biogenic iron, the magnetic characteristics of the samples were measured in the form of isothermal hysteresis curves at a temperature of 2 K (−271.15 °C) and at a magnetic field up to 7 T once the saturation magnetization (*M_S_*) was reached. *M_S_* is the parameter for determining the relative amount of magnetic compounds in the biological samples, of which iron is a dominant component [44,45,47]. Results are expressed as *Ms* (memu/g of dried weight). For determination of magnetic characteristics of ION-originated iron in tissues, samples were prepared as described above, but the magnetic characteristics were measured at temperature 300 K (26.85 °C) in a magnetic field up to 1 T. ION-originated iron content was calculated as described previously [32,39] and expressed as µg of iron per g of dried weight of the tissue. The magnetic properties of the tissues and iron contents were determined in 5–6 rats per group.

### 2.6. Determination of Gene Expression

The relative mRNA expression of *Nfe2l2*, *Pparg*, inducible NOS (*Nos2*), endothelial NOS (*Nos3*), superoxide dismutase 1 (*Sod1*), superoxide dismutase 2 (*Sod2*), glutathione peroxidase 4 (*Gpx4*), *Hamp*, *Fth1*, *Fpn1*, *Tf*, *Tfr1*, *Dmt1*, *Irp1* and β-actin (*Actb*, as housekeeping gene) were determined by using a two-step reverse transcription quantitative polymerase chain reaction (RT-qPCR).

The total RNA of the tissue samples was isolated by using the PureZOL™ RNA Isolation Reagent (Bio-Rad, Hercules, CA, USA) according to the manufacturer’s protocols. The amount of total isolated RNA was spectrophotometrically quantified at 260/280 nm and 260/230 nm by using a NanoDrop spectrophotometer (Thermo Scientific, Waltham, MA, USA). In the next step, the isolated RNA was reverse transcribed into cDNA by using Eppendorf Mastercycler (Eppendorf AG, Elbmarsch, Germany) and the reaction mixture of iScript™ cDNA Synthesis Kit (Bio-Rad, Hercules, CA, USA) according to the manufacturer’s instructions. Gene amplification by using qPCR was performed on a CFX96 Real-Time PCR detection system (Bio–Rad, Hercules, CA, USA). SsoAdvanced Universal SYBR Green Supermix (Bio-Rad, Hercules, CA, USA) was used for gene amplification.

The PCR mixture contained 1.5 μL of five-fold diluted template cDNA, 10 μL SsoAdvanced mix, 1.5 μL of both forward and reverse primers (Metabion, Germany, 4 μmol/L) and 5.5 μL of nuclease-free water (Sigma–Aldrich, Germany) in a final volume of 20 μL. The thermal cycling conditions were as follows: (1) 50 °C for 1 min, (2) 95 °C for 30 sec, (3) 39 cycles consisting of (a) 95 °C for 10 sec, (b) annealing temperature (depending on the selected primer (see Table 1) for 30 sec and (4) melt curve analysis. Determination of melt curves for amplicon analyses was constructed at 59–95 °C, 5 sec/1 °C. Samples were measured using Bio–Rad CFX Manager software (version 2.0). Expression of studied genes was expressed as the ratio of gene expression with respect to β-actin levels (*Actb*). Gene-specific primers were designed using the PubMed database (Gene) and program (Primer-BLAST). The following pairs of primer sequences, melting temperatures, accession number and amplicon size of studied genes are described in Table 1. The plates with samples were measured with CFX96 Touch™ Real Time-PCR Detection System (Bio-Rad, Hercules, CA, USA); the program started with heating up the plate to 50 °C (2 min), then increasing the temperature to 95 °C (30 s). The program continued with 39 cycles repeating the steps of denaturation (95 °C, 10 s) and annealing (30 s). The fluorescent dye SYBR Green (Bio-Rad, Hercules, CA, USA) was used for the detection of PCR products. The signal intensity during amplification was detected with Bio-Rad CFX Manager software. The gene expression is presented as the ratio of gene expression to *Actb*.

## 3. Results

### 3.1. MAP, HR and Plasma Corticosterone Levels

**Figure 1:**
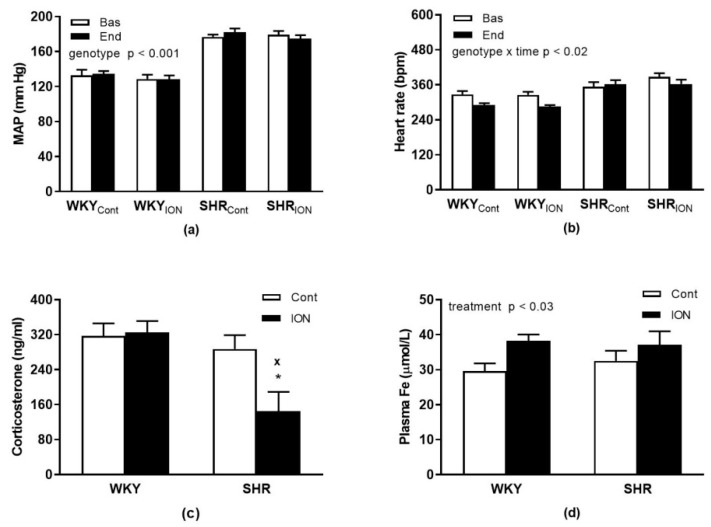
Mean arterial blood pressure (**a**), heart rate (**b**), plasma corticosterone (**c**) and plasma iron levels (**d**). The results are the mean ± SEM. * *p* < 0.01 vs. control SHR, ^x^ *p* < 0.03 vs. ION-treated WKY group. Abbreviations: MAP, mean arterial blood pressure; Bas, basal levels; End, levels determined 100 min post-infusion; SHR, spontaneously hypertensive rats; WKY, Wistar-Kyoto rats; Cont, control group; ION, iron oxide nanoparticle-treated group.

The basal MAP of WKY and SHR rats were 130 ± 4 and 178 ± 3 mmHg, respectively (genotype: F_(1,21)_ = 136.2, *p* < 0.0001). The MAP at the end of the experiment (i.e., 100 min post-infusion) did not change significantly in any group of rats (Figure 1a).

The basal HR of WKY and SHR rats were 327 ± 7 and 370 ± 11 bpm, respectively (F_(1,21)_ = 27.8.2, *p* < 0.0001). There was a significant interaction of genotype and time for HR (F_(1,21)_ = 6.6, *p* < 0.02) with a significantly lower final (i.e., 100 min post-infusion) HR in WKY rats compared to their basal levels (Figure 1b), which was not seen in SHR.

There were no significant differences in plasma corticosterone levels between genotypes (F_(1,20)_ = 3.6, *p* = 0.07). Interaction of genotype and treatment (F_(1,20)_ = 7.3, *p* < 0.02) revealed a significant decrease of plasma corticosterone in ION-treated SHR (SHR_ION_) vs. control SHR (SHR_Cont_) and ION-treated WKY (WKY_ION_, Figure 1c). Determination of total iron content in plasma of rats revealed a significant effect of treatment (F_(1,20)_ = 5.6, *p* < 0.03), with higher levels in ION-treated rats vs. controls (Figure 1d).

### 3.2. Nitric Oxide Synthase Activities and Superoxide Productions

**Figure 2:**
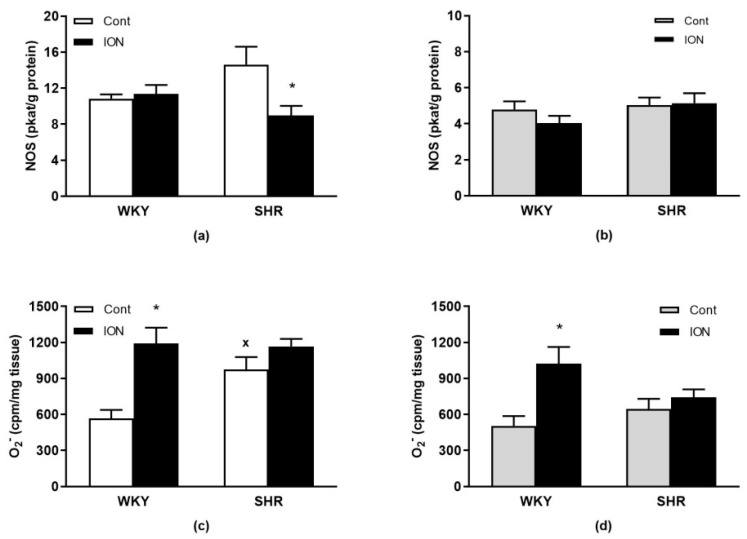
Nitric oxide synthase activity in the liver (**a**) and left heart ventricle (**b**). Superoxide production in the liver (**c**) and left heart ventricle (**d**). The results are the mean ± SEM, * *p* < 0.04 vs. the control group of the same genotype, ^x^ *p* < 0.05 vs. control WKY, n = 6/group. Abbreviations: NOS, nitric oxide synthase; cpm, counts/min; for other abbreviations see the legend to Figure 1.

Regarding NOS activity, two-way ANOVA revealed significant interaction of genotype and treatment in the liver (F_(1,21)_ = 5.5, *p* < 0.03) with significantly lower values of NOS activity in ION-treated SHR vs. control SHR (Figure 2a). No changes in NOS activities were found in the LHV (Figure 2b).

For superoxide productions, there were significant interactions of genotype and treatment in both the liver (F_(1,20)_ = 4.9, *p* < 0.04) and the LHV (F_(1,20)_ = 4.7, *p* < 0.05) with significantly higher levels found in ION-treated WKY compared to control WKY (Figure 2c,d). Higher superoxide production was found in the liver of control SHR vs. control WKY (Figure 2c). However, no changes in superoxide productions were found in ION-treated SHR vs. control SHR in both the liver and LHV (Figure 2c,d).

### 3.3. Relative Contents of Biogenic Iron and ION-Originated Iron

**Figure 3:**
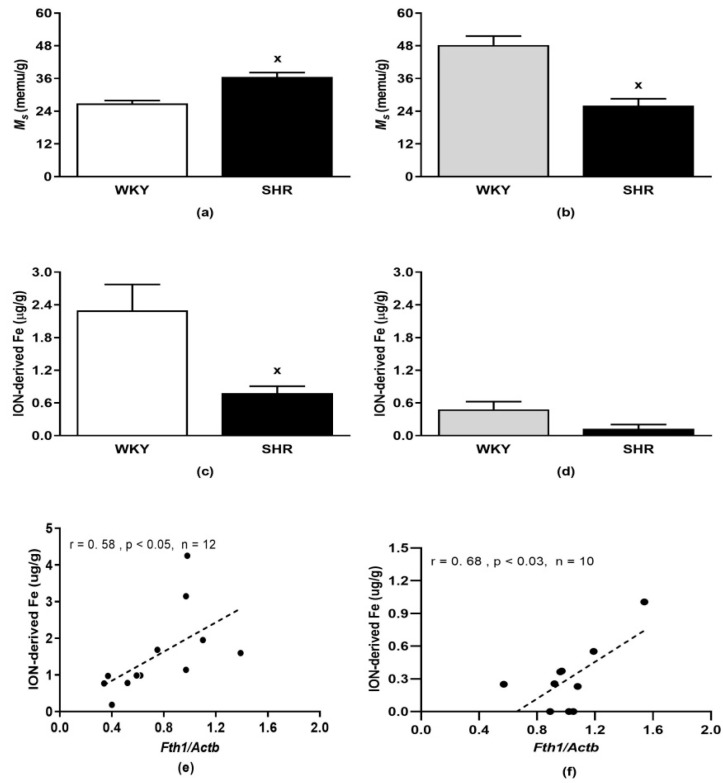
Saturation magnetization in the liver (**a**) and left heart ventricle (**b**). Iron oxide nanoparticle-originated iron content in the liver (**c**) and left heart ventricle (**d**). Pearson correlations between ION-originated iron and *Fth1* gene expressions in the liver (**e**) and left heart ventricle (**f**). The results are the mean ± SEM, ^x^ *p* < 0.04 vs. the WKY group, n = 5–6/group. Abbreviations: *Ms*, saturation magnetization; ION, iron oxide nanoparticles; *Fth1*, ferritin gene; *Actb*, β-actin gene. For other abbreviations see the legend to Figure 1.

Saturation magnetization *Ms* (relative parameter of biogenic iron content) of the liver of control SHR was significantly higher than in WKY (*p* < 0.001, Figure 3a). In contrast, the saturation magnetization of the LHV of SHR was reduced compared to WKY (*p* < 0.001, Figure 3b). ION-originated Fe content was significantly lower in the liver (*p* < 0.02) and the decreasing tendency was also in the LHV (*p* = 0.06, n = 10) of SHR compared to that in the respective WKY value (Figure 3c,d).

In the liver and LHV of ION-treated rats, the amount of ION-originated iron correlated positively with the *Fth1*: r = 0.58, *p* < 0.05 in the liver (Figure 3e) and r = 0.68, *p* < 0.03 in LHV (Figure 3f).

### 3.4. Expression of Genes Involved in Iron Metabolism, Antioxidant Defence and Nitric Oxide Production

**Figure 4:**
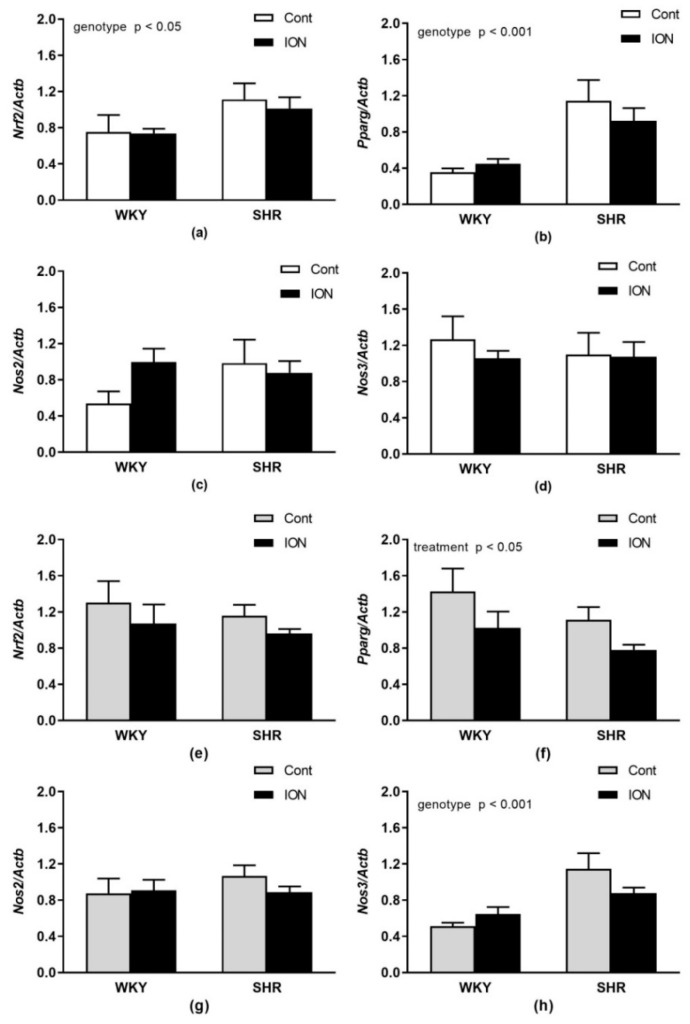
Relative mRNA expressions of nuclear transcription factor *Nfe2l2* (**a**,**e**), *Pparg* (**b**,**f**) and genes involved in nitric oxide production *Nos2* (**c**,**g**) and *Nos3* (**d**,**h**) in the liver (**a**–**d**) and left heart ventricle (**e**–**h**). The values represent the mean ± SEM. See Results section for detailed results of 2-way ANOVA analysis. Abbreviations: *Nfe2l2*, nuclear factor (erythroid-derived 2)-like 2; *Pparg,* peroxisome proliferator-activated receptor gamma; *Nos2*, inducible nitric oxide synthase; *Nos3*, endothelial nitric oxide synthase. Other abbreviations are the same as in Figure 1.

Relative mRNA expressions of *Nfe2l2*, *Pparg, Nos2* and *Nos3* in the liver and LHV are shown in Figure 4. There were significant main effects of genotype for *Nfe2l2* (F_(1,20)_ = 4.59, *p* < 0.05, Figure 4a) and *Pparg* (F_(1,20)_ = 16.23, *p* < 0.001, Figure 4b) in the liver and for *Nos3* (F_(1,20)_ = 17.96, *p* < 0.001, Figure 4h) in the LHV, with higher levels found in SHR. A significant effect of ION treatment was found only for *Pparg* (F_(1,20)_ = 4.59, *p* < 0.05) in the LHV with lower levels in ION-treated rats compared to controls (Figure 4f).

**Figure 5:**
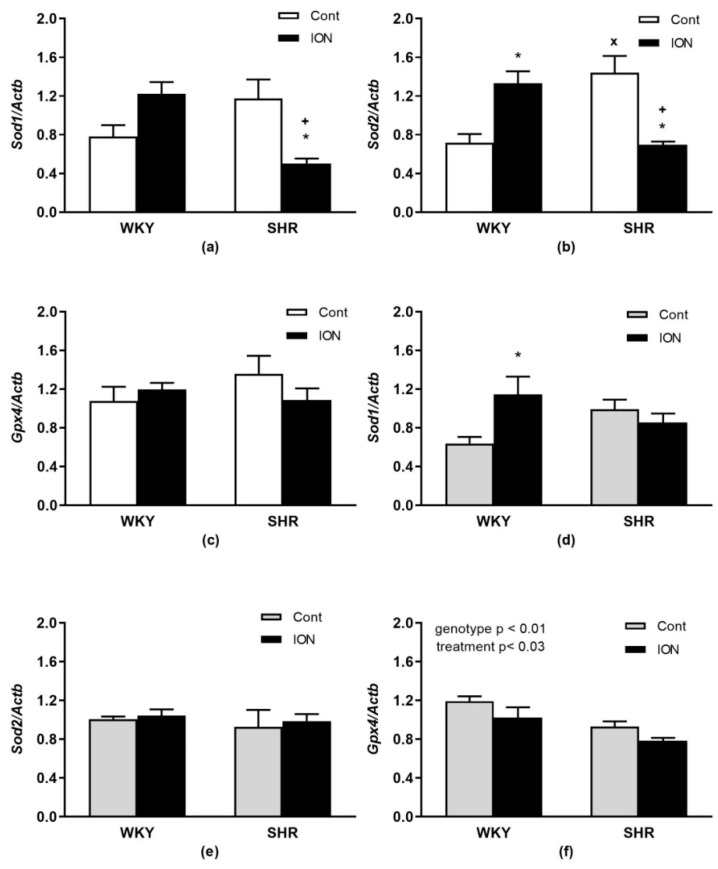
Gene expressions of selected enzymes involved in antioxidant defence *Sod1* (**a**,**d**), *Sod2* (**b**,**e**) and *Gpx4* (**c**,**f**) in the liver (**a**–**c**) and left heart ventricle (**d**–**f**). The values represent the mean ± SEM. * *p* < 0.03 vs. the control group of the same genotype, ^+^ *p* < 0.02 vs. ION-treated WKY group, ^x^ *p* < 0.01 vs. WKY control group, n = 6/group. See Results section for detailed results of 2-way ANOVA analysis. Abbreviations: *Sod1*, superoxide dismutase 1; *Sod2*, superoxide dismutase 2; *Gpx4*, glutathione peroxidase 4. Other abbreviations are the same as in the Figure 1.

Relative mRNA expressions of genes encoding antioxidant enzymes *Sod1, Sod2* and *Gpx4* in the liver and LHV are shown in Figure 5. In the liver, there were significant interactions of genotype and treatment for *Sod1* (F_(1,20)_ = 15.37, *p* < 0.001, Figure 5a) and *Sod2* (F_(1,20)_ = 28.55, *p* < 0.0001, Figure 5b). In the LHV, significant interactions of genotype and treatment were found only for *Sod1* (F_(1,20)_ = 7.20, *p* < 0.02, Figure 5d); no effects were found for *Sod2* (Figure 5e). The mRNA expressions of *Gpx4* were unchanged in the liver (Figure 5c), but in the LHV, significant effects of genotype (F_(1,20)_ = 14.71, *p* < 0.01) and treatment F_(1,20)_ = 5.7, *p* < 0.03, Figure 5f) were found, with lower levels in SHR vs. WKY and for ION-treated rats vs. controls.

**Figure 6:**
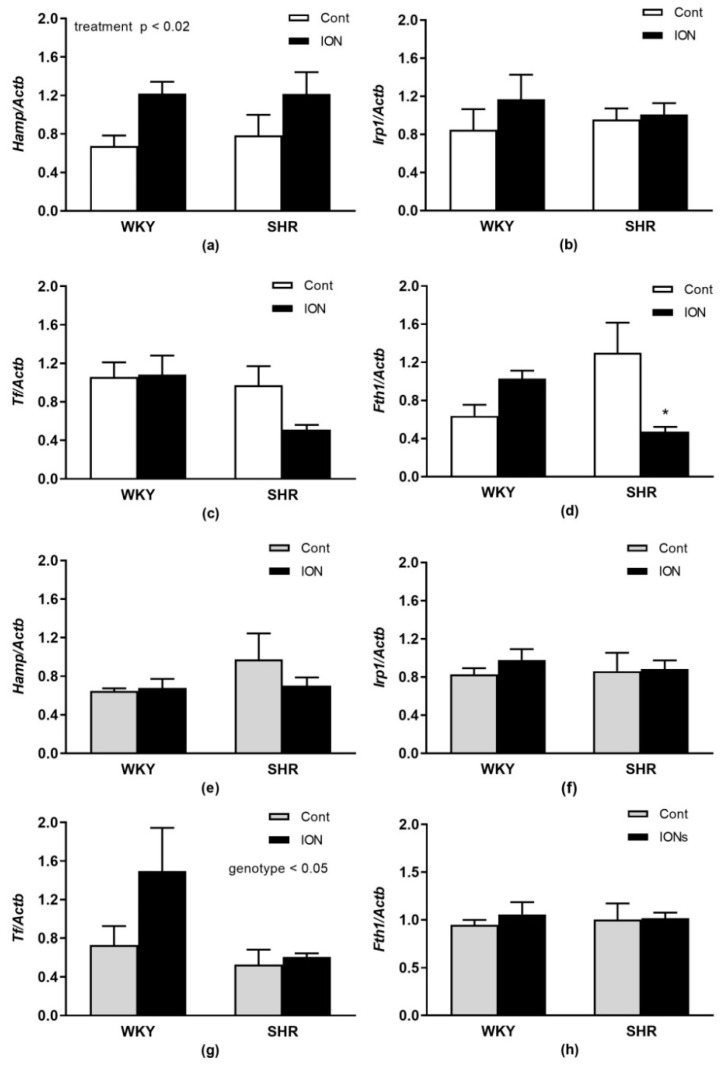
Gene expressions of selected genes associated with iron metabolism regulation or iron carrying and storage in the liver (**a**–**d**) and left heart ventricle (**e**–**h**). The values represent the mean ± SEM. * *p* < 0.03 vs. the control group of the same genotype, n = 6/group. See Results section for detailed results of 2-way ANOVA analysis. Abbreviations: *Hamp*, hepcidin; *Irp1*, iron regulatory protein 1; *Fth1*, ferritin heavy chain 1; *Tf*, transferrin. Other abbreviations are the same as in Figure 1.

In Figure 6, relative mRNA expressions of genes involved in iron metabolism regulation or iron carrying and storage are depicted. A significant effect of ION treatment was found only for *Hamp* (F_(1,20)_ = 6.74, *p* < 0.02) in the liver with higher levels in ION-treated rats compared to controls (Figure 6a). Significant interactions of genotype and treatment in the liver were found only for *Fth1* (F_(1,20)_ = 9.06, *p* < 0.01, Figure 6d); there was a significant decrease in *Fth1* mRNA in ION-treated SHR vs. control SHR. In the LHV, there was a significant reduction in *Tf* gene expression in SHR vs. WKY (effect of genotype, F_(1,20)_ = 4.58, *p* < 0.05, Figure 6g). No differences were found in the gene expression of *Irp1* and *Tf* in the liver (Figure 6b,c) and in the *Hamp*, *Irp1* and *Fth1* in the LHV (Figure 6e,f,h). In the liver, *Hamp* gene expression correlated positively with plasma iron levels (r = 0.58, *p* < 0.006, n = 21), which was not found in the LHV.

**Figure 7:**
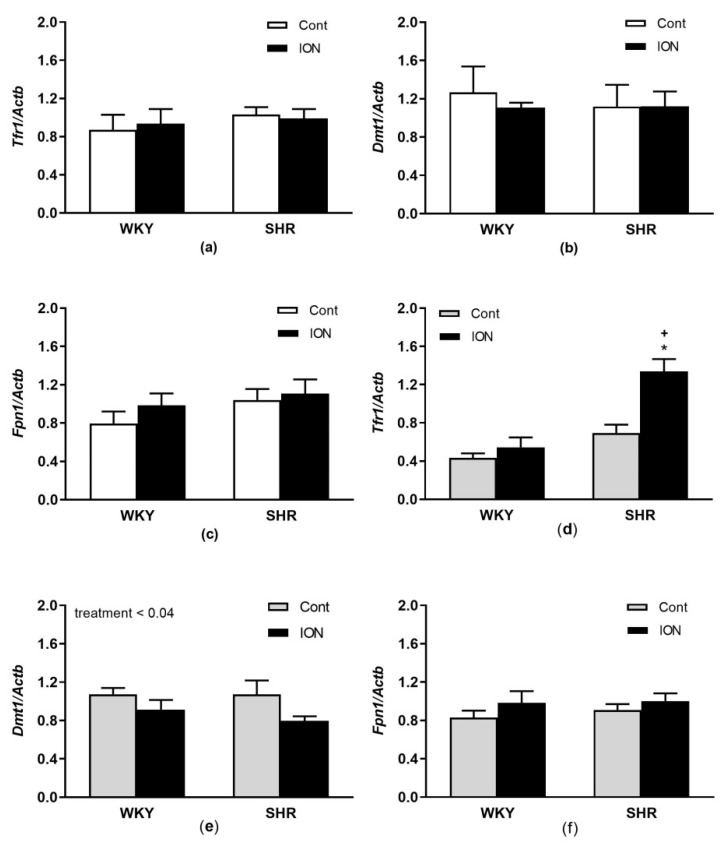
Gene expressions of selected genes associated with iron transport in the liver (**a**–**c**) and left heart ventricle (**d**–**f**). The values represent the mean ± SEM. * *p* < 0.03 vs. the control group of the same genotype, ^+^ *p* < 0.02 vs. ION-treated WKY group, n = 6/group. See Results section for detailed results of 2-way ANOVA analysis. Abbreviations: *Tfr1*, transferrin receptor 1; *Dmt1*, divalent metal transporter 1; *Fpn 1*, ferroportin 1. Other abbreviations are the same as in Figure 1.

In Figure 7, relative mRNA expressions of genes involved in iron transport are depicted. No genotype and/or treatment-related changes in *Tfr1*, *Dmt1* and *Fpn1* gene expressions were found in the liver (Figure 7 a–c). In the LHV, a significant effect of genotype (F_(1,20)_ = 30.02, *p* < 0.0001), treatment (F_(1,20)_ = 15.27, *p* < 0.001) and interactions of genotype and treatment were found for *Tfr1* (F_(1,20)_ = 757, *p* < 0.02, Figure 7d). A significant effect of treatment was found in the LHV for *Dmt1* (F_(1,20)_ = 5.03, *p* < 0.04, Figure 7e). No changes were found in the gene expression of *Fpn1* in the LHV (Figure 7f). The correlations among the genes are in the Supplementary Table S1.

## 4. Discussion

Superparamagnetic iron oxide nanoparticles of γ-Fe_2_O_3_ (maghemite) and Fe_3_O_4_ (magnetite) can be used in various biomedical applications. The advantage of such NPs lies in the possibility to use them for targeted drug delivery in the presence of the magnetic field. However, it is also very important to know the pharmacokinetics of the NPs and their effect on the living organism because iron is an essential biogenic element. In our previous studies, we confirmed the superparamagnetism of IONs used in this study [39] as well as their presence in different tissues depending on the blood pressure [13,32,39,40].

The main findings of this study are that ION-originated iron was found in the livers and hearts of normotensive and hypertensive rats, but considerably less in the tissues of hypertensive SHR than normotensive WKY. ION-originated iron content found in the hearts was roughly around 20% of that determined in the liver of the given genotype. In addition, our study showed considerable tissue differences in the expression of genes involved in iron metabolism, as well as in *Sod* and *Nos* expression, in the livers and hearts. Regarding genotype differences, we found that elevated ION content in the tissues of WKY led to elevated superoxide production, which was not seen in SHR. Lower ION content in the tissues of SHR was associated with reduced plasma corticosterone, reduced total NOS activity in the liver as well as with reduced liver expressions of *Sod1*, *Sod2* and *Fth1*. On the other hand, in WKY, *Sod2* was elevated in the liver and *Sod1* in the heart in ION-treated rats. Despite these genotype- and tissue-related differences, positive correlations were found between plasma iron concentration and hepatic *Hamp* expression as well as between ION-originated iron content and *Fth1* gene expressions in both tissues investigated, suggesting the considerable influence of ION-derived iron on intracellular iron metabolism in these tissues when all rats were taken into account.

Previously we showed that hypertension (i.e., chronic high BP) was associated with an increased incorporation of IONs into the liver and vascular wall of SHR when determined 24 h after the second ION infusion at the dose of 2 mg Fe/kg/day [13]. On the other hand, an acute stress-induced increase in BP in WKY rats treated with a single infusion of IONs (1 mg Fe/kg) led to a decrease in ION-derived iron in circulation and in the liver when determined 100 min post-ION infusion [32]. Those studies showed that high BP, either chronic or acute, is an important factor affecting ION deposition in the liver. In this study, we focused on the heart and liver after a single ION infusion in normotensive and hypertensive rats, which is relevant to the situation of patients with various levels of BP.

We found that a single ION infusion in rest conditions did not induce significant changes in BP and HR in SHR despite an ION-induced decrease in NO production in the liver of SHR (vs. control SHR) and elevated superoxide production in both tissues investigated in WKY. Unchanged BP after ION administration suggests that mild changes in the above-mentioned parameters as well as the increase in overall hepatic iron content due to the incorporation of ION-derived iron had no effect on BP regulation in any rat strain. This finding corresponds with the results of Seravalle et al., who also found no changes in BP in hypertensive patients with hepatic iron overload compared to hypertensive individuals [9]. Surprisingly, we found a significant decrease in corticosterone levels after ION infusion in SHR, suggesting that IONs can interfere with hypothalamic–pituitary–adrenal axis function, specifically under conditions of hypertension, though the exact mechanisms need to be further studied. In the present study, we did not determine iron content in the adrenal glands, but there are reports in humans showing enhanced acute accumulation of iron nanoparticles within the adrenals that was comparable to that in the tissues of the reticuloendothelial system, such as the liver and spleen [48]. In spite of missing data in hypertensive patients, our study demonstrates that the adrenals of hypertensive individuals may display increased sensitivity to ION administration.

Our study also revealed that plasma iron concentrations were elevated in both rat genotypes after ION infusion. However, plasma iron levels do not reflect ION-originated iron content in the tissues. Using SQUID magnetometry, we found elevated *M_S_*, an indirect but very sensitive parameter, reflecting total iron content [39] in the liver of control SHR compared to control WKY. Despite that the liver is considered a rich source of iron in the organism, high levels of iron are also in the heart, in which iron metabolism has to be regulated very precisely to prevent cardiac dysfunction [49]. In addition, SQUID magnetometry allows us to determine the amount of ION-originated iron, which is not possible to distinguish from biogenic iron present in the tissues by biochemical or histological methods [39]. We found that, in conditions of this study, the content of ION-originated iron was lower in the hearts than in the livers and also in tissues of ION-treated SHR compared to the ION-treated WKY. In fact, in three of five hearts of ION-treated SHR we did not find ION-originated iron. However, in both tissues, positive correlations were found between ION-originated iron and *Fth1* gene expressions, suggesting the significant influence of ION-originated iron on innate iron metabolism.

This effect was also confirmed by elevated *Hamp* expression in the liver of ION-treated rats and by a positive correlation between plasma iron and *Hamp* gene expression in the liver. It is known that the hepatic release of hepcidin is regulated predominantly by intrahepatic iron concentration [50]. However, hepcidin can also be synthesized in smaller amounts by the other tissues, including the heart [51] in which it participates in the regulation of local iron levels [52]. Recent studies show that hepcidin has an irreplaceable role in regulating iron homeostasis in the heart; hepcidin deficiency results in cardiac dysfunction, left ventricular hypertrophy and apoptosis [53]. In our study, no changes in cardiac *Hamp* expression were found, suggesting that the heart functions should not be considerably altered by ION-originated iron after a single low-dose ION infusion. However, IONs and/or iron released after nanoparticle disintegration can enter redox processes and induce oxidative stress [54,55]. Here we found elevated superoxide production only in the tissues of WKY, which is in agreement with the higher ION-originated iron content found in the tissues of WKY. In addition, *Sod2* was elevated in the liver and *Sod1* in the heart of WKY, while both *Sod1* and *Sod2* were significantly reduced in the liver of ION-treated SHR vs. control SHR. Despite our expectation, no corresponding changes (regarding *Sod* expressions) were found in the expression of the *Nfe2l2* gene in both tissues investigated. On the other hand, we found positive correlations between *Nfe2l2* and *Nos2*, *Nos3*, *Dmt1*, *Gpx4, Sod2* and *Pparg* in the liver. No such correlations with *Nfe2l2* were found in the heart, except for that with *Pparg*, which correlated positively with *Nfe2l2* in both tissues investigated. This study also showed two interesting relations among the gene expressions in the liver and heart when all rats were taken into account. Firstly, Of the 24 correlations found among the genes investigated in this study in the liver, only 1 was negative. In the heart, of the 39 correlations found among the genes investigated, 17 were negative and 22 were positive. Secondly, all correlations with *Dmt1* or *Fth1* were positive in the liver and only two genes (*Fpn1* and *Tfr1*) correlated positively with *Irp1*. In contrast, in the heart, all genes which correlated positively with *Fth1* or *Irp1*, correlated negatively with *Dmt1*. These findings suggest considerable differences in the expression of genes involved in iron metabolism in the liver and heart. Since the optimal iron concentration in the heart is critical for normal cardiac function [49], we assume that the sensitivity of cardiomyocytes to iron is higher compared to that in the liver. Rapid, but not necessarily big, changes in the expression of various genes and corresponding proteins can maintain optimal intracellular iron concentrations in the heart, specifically under oxidative and/or nitrosative stress, as both ROS and NO participate in the regulation of iron metabolism [56,57]. In the heart, we also found a negative correlation between the *Fth1* and *Dmt1* genes, supposedly under the control of *Irp1*, which suggests the finely tuned regulation of intracellular iron concentration. In addition, opposite correlations of the *Fth1* and *Dmt1* genes with other genes mentioned in the supplementary Table S1, especially with *Nos* and *Sod* genes, can result in the increase in antioxidant defence and reduction of NO production. NO, in addition to its role in cardiovascular functions [58,59], has an important role in iron metabolism by modulation of cytosolic aconitase (ACO1)/IRP1 function [60]. In conditions of low levels of intracellular iron, the apo form of ACO1 has the function of IRP1, while at high iron levels, its holo form containing the [4Fe-4S]^2+^ cluster possesses ACO1 enzymatic activity [61]. NO can promote the release of iron from the Fe-S centres of ACO1 and thus induce the IRP1 function of this bifunctional protein [61]. High levels of IRP1 protein support an increase of *Fth1* mRNA translation resulting in elevated iron storage capacity in ferritin cores. In addition, high levels of IRP1 decrease *Dmt1* and *Tfr1* mRNA stability, which leads to a reduced influx of iron into the cell [62]. Surprisingly, in this study, there were no correlations with the *Nfel2l* and *Pparg* genes and other genes investigated in the heart, which is in contrast to the liver. In the liver, the antioxidant genes (*Sod2*, *Gpx4*) and *Nos2* and *Nos3* genes correlated positively with *Nfel2l*, in agreement with previous findings of elevated NRF2 translocation into the nucleus in the presence of ROS and NO [20,21]. Interestingly, none of the genes investigated in the liver correlated with hepatic *Hamp* and only two genes (*Fpn1*, *Tfr1*) correlated with *Irp1* expression. Collectively, the results suggest that while changes observed on the gene levels in the heart seem to be related to changes in iron load, changes in the liver seem to be more related to oxidative stress and/or NO, which may be induced by altered hepatic iron content. Indeed, superoxide production was elevated in the liver of ION-treated WKY as well as in both SHR groups compared to control WKY, which was not found in the heart. Thus, our data showed considerable differences in the expression of genes involved in iron metabolism in the heart and the liver of rats. In agreement, a recent study by Kasztura et al. [63] has shown that the mechanisms of iron uptake, storage and clearance differ between the liver and heart in the porcine model of heart failure progression.

## 5. Conclusions

Our results showed that a single administration of IONs intravenously at a relatively low dose was found in the liver and the heart of both rat genotypes 100 min after their infusion. Lower content of ION-derived iron was found in the tissues of SHR compared to WKY as well as in the hearts compared to livers, confirming the hypothesis that high blood pressure significantly alters the biodistribution of IONs with less incorporation into the heart than into the liver. In addition, our results showed differences in the regulation of iron metabolism on gene levels in the heart and liver. In the heart, expressions of various genes involved in iron metabolism, including *Sod* and *Nos*, seem to be mediated predominantly by iron content and regulated by IRP1-mediated mechanisms. In the liver, the expression of genes correlated with *Nfe2l2,* suggesting a predominant effect NRF2–mediated mechanisms in the regulation of iron metabolism due to an altered redox balance and NO bioavailability.

## Figures and Tables

**Table 1:** Primer pairs used to amplify selected genes.

Gene	Primer Pairs	Tm (°C)	Amp (bp)
*Nos3*	Forward: GAT CCC CCG GAG AAT GGA GA	60	105
(NM_021838.2)	Reverse: TCG GAT TTT GTA ACT CTT GTG CT		
*Nos2*	Forward: AAA CGC TAC ACT TCC AAC GC	59	91
(NM_012611.3)	Reverse: TGC TGA GAG CTT TGT TGA GGT C		
*Nfe2l2*	Forward: TGC CAT TAG TCA GTC GCT CTC	60	102
(NM_031789.2)	Reverse: ACC GTG CCT TCA GTG TGC		
*Pparg*	Forward: CTC ACA ATG CCA TCA GG TTT GG	59	84
(NM_013124.3)	Reverse: GCT GGT CGA TAT CAC TGG AGA T		
*Sod1*	Forward: CTG AAG GCG AGC ATG GGT TC	60	131
(NM_017050.1)	Reverse: TCC AAC ATG CCT CTC TTC ATC C		
*Sod2*	Forward: GCT GGC CAA GGG AGA TGT TAC	60	83
(NM_017051.2)	Reverse: TGC TGT GAT TGT ATG GCC CC		
*Gpx4*	Forward: TAA GTA CAG GGG TTG CGT GTG	60	135
(NM_001368043.1)	Reverse: CAA GGG AAG GCC AGG ATT CG		
*Dmt1 (Slc11a2)*	Forward: CTA CTT GGG TTG GCA GTG TTT G	60	94
(NM_013173.2)	Reverse: ATC TTC GCT CAG CAG GAC TTT		
*Tf*	Forward: GCT CCG AAC AAC AGA GAG GG	60	126
(NM_001013110.1)	Reverse: AGC AGT GTT CTT TCC GTT CGT		
*Tfr1*	Forward: GCT ATG AGG AAC CAG ACC GC	58	78
(NM_022712.1)	Reverse: CAC TGG ACT TCG CAA CAC CA		
*Fth1*	Forward: ACG TCT ATC TGT CCA TGT CTT GTT	60	149
(NM_012848.2)	Reverse: GAA GAT TCG TCC ACC TCG CT		
*Fpn1 (Slc40a1)*	Forward: GAC CTC ACC TAA AGA TAC TGA GCC	59	130
(NM_133315.2)	Reverse: GAA GGG TTC TGC GAT CTG GG		
*Irp1 (Aco1)*	Forward: ACG TCA AAA CCA GCC TGT CT	59	100
(NM_017321.1)	Reverse: ACC ACG TCA AAC CCT AAC TGG		
*Hamp (Hepc)*	Forward: CTA TCT CCG GCA ACA GAC GAG	60	110
(NM_053469.1)	Reverse: TGT CTC GCT TCC TTC GCT TC		
*Actb*	Forward: CTC TGT GTG GAT TGG TGG CT	59	139
(NM_031144.3)	Reverse: CGC AGC TCA GTA ACA GTC CG		

Abbreviations: *Nos3*, endothelial nitric oxide synthase; *Nos2*, inducible nitric oxide synthase; *Nfe2l2*, nuclear factor (erythroid-derived 2)-like 2; *Pparg*, peroxisome proliferator-activated receptor gamma; *Sod1*, superoxide dismutase 1; *Sod2*, superoxide dismutase 2; *Gpx4*, glutathione peroxidase 4; *Dmt1*, divalent metal transporter; *Tf*, transferrin; *Tfr1*, transferrin receptor; *Fth1*, ferritin heavy chain 1; *Fpn*, ferroportin; *Irp1* (*Aco1*), iron-regulatory protein 1 or aconitase 1; *Hamp*, hepcidin; *Actb*, β-actin; Tm, metlting temperature; Amp, amplicon; bp, base pare of DNA.

## Data Availability

The data presented in this study are available on request from the corresponding author.

## Supplementary Materials

The following supporting information can be downloaded at https://www.mdpi.com/article/10.3390/pharmaceutics15051475/s1. Table S1: Correlations of the gene expressions in the livers and left heart ventricles of rats.
