# Supplementary material for: A Single Infusion of Polyethylene Glycol-Coated Superparamagnetic Magnetite Nanoparticles Alters Differently the Expressions of Genes Involved in Iron Metabolism in the Liver and Heart of Rats
Source: Pharmaceutics. 2023 May 12;15(5):1475. doi: 10.3390/pharmaceutics15051475 (PMC10220547; doi:10.3390/pharmaceutics15051475)
Supplement: Supplementary file 1 [file pharmaceutics-15-01475-s001.zip › pharmaceutics-2401161-supplementary.pdf]

**A single infusion of polyethylene glycol-coated superparamagnetic magnetite nanoparticles alters differently the expressions of genes involved in iron metabolism in the liver and heart of rats**

Michal Kluknavsky[1x], Andrea Micurova[1x], Martin Skratek[2], Peter Balis[1], Monika Okuliarova[3], Jan Manka[2], Iveta Bernatova[1*]

**Table S1. Correlations of the gene expressions in the livers and left heart ventricles of rats**

## Abbreviations

*LHV:* left heart ventricle, *Nos3*: endothelial nitric oxide synthase, *Nos2*: inducible nitric oxide synthase, *Nfe2l2*: nuclear factor (erythroid-derived 2)-like 2, *Pparg*: peroxisome proliferator-activated receptor gamma, *Sod1*: superoxide dismutase 1, *Sod2*: superoxide dismutase 2, *Gpx4:* glutathione peroxidase 4, *Dmt1*: divalent metal transporter, *Tf*: transferrin, *Tfr1*: transferrin receptor, *Fth1*: ferritin heavy chain 1, *Fpn*: ferroportin, *Irp1 (Aco1)*: *iron-regulatory protein 1 or aconitase 1, Hamp*: hepcidin.

| **Liver n = 24** | ***Fth1*** | ***Dmt1*** | ***Tfr1*** | ***Tf*** | ***Fpt*** | ***Irp1*** | ***Hamp*** | ***Gpx4*** | ***Sod2*** | ***Sod1*** | ***Nos2*** | ***Nos3*** | ***Pparg*** | | **LHV n = 24** |
|---|---|---|---|---|---|---|---|---|---|---|---|---|---|---|---|
| ***Nfe2l2*** | | $r = 0.76$<br>$p < 0.0001$ | | | | | | $r = 0.73$<br>$p < 0.0001$ | $r = 0.52$<br>$p < 0.001$ | | $r = 0.68$<br>$p < 0.0003$ | $r = 0.77$<br>$p < 0.0001$ | $r = 0.84$<br>$p < 0.0001$ | | |
| ***Pparg*** | | $r = 0.51$<br>$p < 0.01$ | | $r = -0.50$<br>$p < 0.02$ | | | | $r = 0.70$<br>$p < 0.001$ | | | $r = 0.73$<br>$p < 0.0001$ | $r = 0.53$<br>$p < 0.01$ | | $r = -0.83$<br>$p < 0.0001$ | ***Dmt1*** |
| ***Nos3*** | | $r = 0.95$<br>$p < 0.0001$ | | | | | | $r = 0.72$<br>$p < 0.0001$ | | | $r = 0.65$<br>$p < 0.001$ | | $r = -0.41$<br>$p < 0.05$ | | ***Tfr1*** |
| ***Nos2*** | | $r = 0.56$<br>$p < 0.005$ | | | | | | $r = 0.80$<br>$p < 0.0001$ | | | | | $r = -0.51$<br>$p < 0.02$ | $r = 0.61$<br>$p < 0.002$ | ***Tf*** |
| ***Sod1*** | $r = 0.84$<br>$p < 0.0001$ | | | $r = 0.67$<br>$p < 0.001$ | | | | | $r = 0.52$<br>$p < 0.01$ | | $r = 0.63$<br>$p < 0.001$ | $r = 0.46$<br>$p < 0.03$ | $r = -0.74$<br>$p < 0.0001$ | $r = 0.71$<br>$p < 0.0001$ | ***Fpt*** |
| ***Sod2*** | $r = 0.47$<br>$p < 0.03$ | | | | | | | | | $r = 0.55$<br>$p < 0.006$ | $r = 0.44$<br>$p < 0.04$ | | $r = -0.69$<br>$p < 0.0003$ | $r = 0.72$<br>$p < 0.0001$ | ***Irp1*** |
| ***Gpx4*** | | $r = 0.61$<br>$p < 0.002$ | | | | | | | | | | | | $r = 0.51$<br>$p < 0.02$ | ***Hamp*** |
| ***Hamp*** | | | | | | | | | | $r = -0.63$<br>$p < 0.0001$ | | $r = -0.52$<br>$p < 0.01$ | $r = 0.70$<br>$p < 0.0002$ | $r = -0.58$<br>$p < 0.004$ | ***Gpx4*** |
| ***Irp1*** | | | $r = 0.72$<br>$p < 0.001$ | | $r = 0.70$<br>$p < 0.001$ | | | $r = 0.61$<br>$p < 0.002$ | $r = 0.52$<br>$p < 0.01$ | | | | $r = -0.52$<br>$p < 0.009$ | $r = 0.57$<br>$p < 0.004$ | ***Sod2*** |
| ***Fpt*** | | | $r = 0.54$<br>$p < 0.01$ | | | | $r = -0.60$<br>$p < 0.002$ | | $r = 0.46$<br>$p<0.03$ | $r = 0.59$<br>$p < 0.003$ | $r = 0.49$<br>$p<0.02$ | | $r = -0.51$<br>$p < 0.02$ | $r = 0.63$<br>$p < 0.001$ | ***Sod1*** |
| ***Tf*** | $r = 0.69$<br>$p < 0.001$ | | | | | | | | $r = -0.54$<br>$p < 0.007$ | $r = -0.41$<br>$p < 0.05$ | $r = -0.44$<br>$p < 0.03$ | | $r = 0.63$<br>$p < 0.002$ | $r = -0.58$<br>$p < 0.004$ | ***Nos2*** |
| ***Tfr1*** | | | | $r = 0.51$<br>$p < 0.02$ | | $r = - 0.56$<br>$p < 0.005$ | | | $r = -0.48$<br>$p < 0.02$ | | | | $r = 0.46$<br>$p < 0.03$ | $r = -0.44$<br>$p < 0.04$ | ***Nos3*** |
| ***Dmt1*** | | | | | | | | | | | | | | | ***Pparg*** |
| | | $r = 0.79$<br>$p < 0.0001$ | | | | | | | | | | | | | ***Nfe2l2*** |
| **Liver n = 24** | | ***Pparg*** | ***eNOS*** | ***iNOS*** | ***Sod1*** | ***Sod2*** | ***Gpx4*** | ***Hamp*** | ***Irp1*** | ***Fpt*** | ***Tf*** | ***Tfr1*** | ***Dmt1*** | ***Fth1*** | **LHV n = 24** |
